# Circulating growth factor concentrations and breast cancer risk: a nested case-control study of IGF-1, IGFBP-3, and breast cancer in a family-based cohort

**DOI:** 10.1186/s13058-020-01352-0

**Published:** 2020-10-22

**Authors:** Kelsey R. Monson, Mandy Goldberg, Hui-Chen Wu, Regina M. Santella, Wendy K. Chung, Mary Beth Terry

**Affiliations:** 1 Department of Epidemiology, Mailman School of Public Health, Columbia University, 722 West 168th Street, New York, NY 10032 USA; 2 Department of Environmental Health Sciences, Mailman School of Public Health, Columbia University, 722 West 168th Street, New York, NY 10032 USA; 3 Herbert Irving Comprehensive Cancer Center, Columbia University Medical Center, 1130 St. Nicholas Avenue, New York, NY 10032 USA; 4 Department of Pediatrics, Columbia University, 622 West 168th Street, New York, NY USA; 5 Department of Medicine, Columbia University Medical Center, 630 West 168th Street, New York, NY USA

**Keywords:** IGF-1, IGFBP-3, Breast cancer, Risk model, Family history

## Abstract

**Background:**

Insulin-like growth factor 1 (IGF-1) and binding protein 3 (IGFBP-3) are associated with breast cancer in women at average risk of cancer. Less is known whether these biomarkers also predict risk in women with breast cancer family history.

**Methods:**

We conducted a nested case-control study within the New York site of the Breast Cancer Family Registry (BCFR, *n* = 80 cases, 156 controls), a cohort enriched for breast cancer family history. Using conditional logistic regression, we estimated the association between IGF-1 and IGFBP-3 levels and breast cancer risk and examined whether this risk differed by predicted absolute breast cancer risk based on pedigree models.

**Results:**

The overall association between IGF-1 or IGFBP-3 elevation (≥ median in controls) and breast cancer risk was elevated, but not statistically significant (IGF-1 OR = 1.37, 95% CI = 0.66–2.85; IGFBP-3 OR = 1.62, 95% CI = 0.81–3.24). Women with elevated predicted absolute 10-year risk ≥ 3.4% and elevated IGFBP-3 (≥ median) had more than a 3-fold increased risk compared to women with lower predicted absolute 10-year risk (< 3.4%) and low IGFBP-3 (OR = 3.47 95% CI = 1.04–11.6).

**Conclusions:**

These data offer some support that the overall magnitude of the associations between IGF-1 and IGFBP3 seen in average risk cohorts may be similar in women enriched with a strong breast cancer family history.

## Background

Insulin-like growth factor (IGF-1) and its primary binding protein (IGFBP-3) are known risk factors for breast cancer due to their ability to stimulate mitosis and suppress programmed cell death [1]. Studies have shown that both breast cancer development and recurrence are associated with IGF-1 and IGFBP-3 levels in women [2, 3]. To date, these studies have been in cohorts comprised of women from the general population who are therefore at average risk of cancer. Women with a family history of breast cancer are two to four times more likely to develop the disease compared to women with no family history [4]. With few exceptions, there exists limited data regarding whether IGF-1/IGFBP-3 biomarkers may be associated with cancer in women at high risk of breast cancer [2]. Most epidemiological cohorts are not powered to address the association between risk factors and breast cancer across the spectrum of predicted absolute breast cancer risk.

## Methods

### Study population

Within the New York site of the Breast Cancer Family Registry (BCFR), we prospectively followed a sub-cohort of women unaffected with breast cancer at study entry. The New York site of the BCFR recruited family members with multiple instances of breast or ovarian cancer to construct a family-based registry to assess risk due to a woman’s familial risk profile (FRP) (for full study details, see [4–6]). In this nested case-control study within the larger New York BCFR cohort, 80 prospectively ascertained invasive breast cancer cases were identified and matched to 156 controls by age, ethnicity, and follow-up time. We determined breast cancer diagnoses through self-report, medical record linkage, or family member report. Cases and controls must have had both breasts at baseline and an age at baseline interview of ≤ 70 years. While the history of prior breast cancer was an exclusion criterion for entry into this unaffected BCFR cohort, both cases and controls could have had prior other non-breast cancers. All women participating in the BCFR provided written informed consent, and the study was approved by the Columbia University Medical Center Institutional Review Board.

### Biomarker assessment

We collected a 30-mL blood sample at baseline, which was processed and stored at the New York site of the BCFR [6]. To assess absolute levels of biomarkers in cases versus controls, we measured IGF-1 and IGFBP-3 concentrations (in ng/mL) in stored plasma at the Irving Institute for Clinical and Translational Research Core Biomarkers Lab at Columbia University. We measured IGF-1 using a chemiluminescent immunoassay (CLIA) on the Immulite 1000 automated platform (Siemens Healthcare Diagnostics, Deerfield, IL). Two subjects in this analysis had IGF-1 levels below 25 ng/mL, the limit of detection of the assay; the IGF-1 value for these women was set to 25 ng/mL. The IGF-1 inter-assay coefficient of variation (CV), which we calculated from a pooled sample, was 4.6%. We measured IGFBP-3 using a Human IGFBP-3 Quantikine enzyme-linked immunosorbent assay (ELISA) kit (R&D Systems, Minneapolis, MN). The IGFBP-3 inter-assay CV from a pooled sample was 4.8%. The laboratory was blinded to case-control status.

### Statistical analysis

We calculated each woman’s 10-year absolute risk of breast cancer using the BOADICEA (Breast and Ovarian Analysis of Disease Incidence and Carrier Estimation Algorithm) risk model [7, 8]. In our analyses, the risk score was analyzed as both a continuous and dichotomized variable to differentiate between women at “average” and “high” risk. A 10-year risk above 3.4% is considered “elevated” per National Comprehensive Cancer Network (NCCN) Clinical Practice Guidelines; therefore, we dichotomized BOADICEA to < 3.4% and ≥ 3.4% [9].

We performed conditional logistic regression on the 1:2 matched sets of cases and controls to estimate the breast cancer risk for women with high circulating IGF-1 or IGFBP-3 biomarker levels compared to women with low levels. We defined elevated biomarkers as greater than or equal to the median value in controls (93.95 ng/mL for IGF-1; 2009 ng/mL for IGFBP-3) and assessed risk for both elevated and continuous log-transformed biomarker levels. To test for the interaction between biomarker levels and BOADICEA score, we first tested for linearity of the biomarker level. We then used a cross-product term to assess multiplicative interaction and examined interaction on the additive scale using relative excess risk due to interaction (RERI). We adjusted for baseline covariates including obesity (body mass index ≥ 30 kg/m^2^), smoking and alcohol consumption, menopausal status, and BOADICEA score.

## Results

Table 1 presents the baseline characteristics of the participants in the nested case-control study compared to the other participants in the unaffected cohort. Participants in this analysis were slightly older (mean age, case-control = 45.8 years; cohort = 42.8 years), less ethnically diverse (% white, case-control = 86.9%; cohort = 65.7%), and at higher risk of breast cancer (mean BOADICEA score, case-control = 6.76%; cohort = 4.88%). Cases and controls were comparable across all covariates except for BOADICEA risk scores, with cases demonstrating higher scores than controls (Table 1). Cases and controls did not have significantly different IGF-1 (geometric mean = 93.9 ng/mL, SD = 1.45 in controls; geometric mean = 95.0 ng/mL, SD = 1.46 in cases) or IGFBP-3 (geometric mean = 1960 ng/mL, SD = 1.32 in controls; geometric mean = 2069 ng/mL, SD = 1.25 in cases) levels (Table 1).

**Table 1:** Baseline characteristics of nested case-control participants by breast cancer status (*n* = 236), all other participants in the full unaffected cohort (*n* = 1729), New York Site of the BCFR

	Cases: women withbreast cancer, *N* = 80	Matched controls: womenwithout breast cancer, *N* = 156	Nested case-controltotal, *N* = 236	Unaffected cohort,*N* = 1729
	*N* (%)	*N* (%)	*N* (%)	*N* (%)
Age at blood draw				
*Mean years (s.d.)*	46.2 (10.3)	45.5 (10.9)	45.8 (10.7)	42.8 (13.2)^b^
Ethnicity				
Non-Hispanic White	71 (88.8)	134 (85.9)	205 (86.9)	1136 (65.7)
Hispanic	9 (11.3)	20 (12.8)	29 (12.3)	414 (23.9)
Others	0 (0)	2 (1.3)	2 (0.9)	179 (10.4)
BMI				
*Mean kg/m*^*2*^ *(s.d.)*	24.6 (4.5)	23.8 (4.4)	24.1 (4.4)	25.2 (5.6)
Menopausal status				
Pre-menopausal	52 (65.0)	112 (71.8)	164 (71.9)	1129 (67.0)
Post-menopausal	27 (33.8)	37 (23.7)	64 (28.1)	557 (33.0)
Smoking status				
Current	6 (7.5)	14 (9.0)	20 (8.5)	153 (8.9)
Former	29 (36.3)	44 (28.2)	73 (31.1)	513 (29.8)
Never	44 (55.0)	98 (62.8)	142 (60.4)	1054 (61.3)
Alcohol consumption				
Current	27 (33.8)	58 (37.2)	85 (36.5)	505 (29.4)
Former	17 (21.3)	24 (15.4)	41 (17.6)	505 (16.7)
Never	36 (45.0)	71 (45.5)	107 (45.9)	943 (54.9)
BOADICEA score				
*Mean (s.d.)*	7.94 (7.2)	5.55 (5.0)	6.76 (6.84)	4.88 (6.1)
Average risk (< 3.4%)	19 (23.8)	56 (35.9)	75 (32.5)	841 (48.6)
Elevated risk (≥ 3.4%)	61 (76.3)	95 (60.9)	156 (67.5)	888 (51.4)
IGF-1 level (ng/mL)				
*Geometric mean (geometric s.d.)*	95.0 (1.46)	93.9 (1.45)	94.3 (1.45)	*N/A*
*Median (IQR)*	99.6 (45.6)	94.0 (45.8)	96.4 (45.9)	*N/A*
< 93.95	34 (42.5)	78 (50.0)	112 (47.5)	*N/A*
Elevated (≥ 93.95)^a^	46 (57.5)	78 (50.0)	124 (52.5)	*N/A*
IGFBP-3 level (ng/mL)				
*Geometric mean (geometric s.d.)*	2069.3 (1.25)	1960.3 (1.32)	1996.6 (1.30)	*N/A*
*Median (IQR)*	2097 (635)	2009 (652)	2057 (638)	*N/A*
< 2009	34 (42.5)	78 (50.0)	112 (47.5)	*N/A*
Elevated (≥ 2009)^a^	46 (57.5)	78 (50.0)	124 (52.5)	*N/A*

*Abbreviations*: *BCFR* Breast Cancer Family Registry, *BMI* body mass index, *BOADICEA* Breast and Ovarian Analysis of Disease Incidence and Carrier Estimation Algorithm, *IGF-1* insulin-like growth factor 1, *IGFBP-3* insulin-like growth factor binding protein 3, *IQR* interquartile range

^a^Biomarker elevation defined as greater than or equal to the median value in controls

^b^Age at interview (blood draw N/A)

The risk of breast cancer was elevated across models with continuous biomarker measures except for the adjusted model for continuous IGF-1 (OR = 0.77, 95% CI 0.26–2.31, Table 2). We also saw increased risk with elevated biomarker levels, with an OR of 1.37 (95% CI 0.66–2.85) in the adjusted model for elevated IGF-1 and an OR of 1.62 (95% CI 0.81–3.24) for elevated IGFBP-3 (Table 2). We also examined the ratio of IGF-1/IGFBP-3, which was not associated with breast cancer risk in our sample (data not shown).

**Table 2:** Breast cancer relative risk by biomarker level, New York Site of BCFR

	Breast cancer relative risk	
	**OR**^**a**^ **(95% CI)**	**OR**^**b**^ **(95% CI)**
**IGF-1**		
Log-transformed continuous IGF-1 (*N* = 236)		
	1.19 (0.52, 2.70)	0.77 (0.26, 2.31)
Median IGF-1 in controls (*N* = 236)		
< 93.95 ng/mL	*Reference*	*Reference*
Elevated (≥ 93.95 ng/mL)	1.46 (0.80, 2.71)	1.37 (0.66, 2.85)
	**OR**^**a**^ **(95% CI)**	**OR**^**c**^ **(95% CI)**
Interaction with BOADICEA risk score (*N* = 231)		
Low IGF-1 (< 93.95), low BOADICEA risk (< 3.4%) (*N* = 27)	*Reference*	*Reference*
Elevated IGF-1 (≥ 93.95), low BOADICEA risk (< 3.4%) (*N* = 48)	2.49 (0.72, 8.63)	2.76 (0.64, 11.9)
Low IGF-1 (< 93.95), high BOADICEA risk (≥ 3.4%) (*N* = 81)	3.23 (0.99, 10.59)	3.74 (0.99, 14.2)
Elevated IGF-1 (≥ 93.95), high BOADICEA risk (≥ 3.4%) (*N* = 75)	3.82 (1.20, 12.15)*	3.92 (1.07, 14.4)*
RERI	− 0.90 (− 4.22, 2.41)	− 1.55 (− 5.80, 2.69)
*p* value for interaction	0.28	0.21
	**OR**^**a**^ **(95% CI)**	**OR**^**b**^ **(95% CI)**
**IGFBP-3**		
Log-transformed continuous IGFBP-3 (*N* = 236)		
	2.76 (0.84, 9.07)	4.90 (0.99, 24.1)
Median IGFBP-3 in controls (*N* = 236)		
< 2009	*Reference*	*Reference*
Elevated (≥ 2009)	1.41 (0.79, 2.51)	1.62 (0.81, 3.24)
	**OR**^**a**^ **(95% CI)**	**OR**^**c**^ **(95% CI)**
Interaction with BOADICEA risk score (*N* = 231)		
Low IGFBP-3 (< 2009), low BOADICEA risk (< 3.4%) (*N* = 28)	*Reference*	*Reference*
Elevated IGFBP-3 (≥ 2009), low BOADICEA risk (< 3.4%) (*N* = 47)	1.52 (0.48, 4.83)	2.08 (0.55, 7.92)
Low IGFBP-3 (< 2009), high BOADICEA risk (≥ 3.4%) (*N* = 81)	2.09 (0.71, 6.13)	2.26 (0.70, 7.25)
Elevated IGFBP-3 (≥ 2009), high BOADICEA risk (≥ 3.4%) (*N* = 75)	3.24 (1.09, 9.63)*	3.47 (1.04, 11.6)*
RERI	0.63 (− 1.50, 2.77)	0.13 (− 2.60, 2.86)
*p* value for interaction	0.97	0.68

*Abbreviations*: *BCFR* Breast Cancer Family Registry, *CI* confidence interval, *IGF-1* insulin-like growth factor 1, *IGFBP-3* insulin-like growth factor binding protein 3, *RERI* relative excess risk due to interaction

^a^Conditional logistic regression, unadjusted

^b^Conditional logistic regression, adjusted for age at blood draw, BMI, smoking status, alcohol consumption, menopausal status, continuous BOADICEA score, and opposing biomarker (IGF-1 or IGFBP-3)

^c^Conditional logistic regression, adjusted for age at blood draw, BMI, smoking status, alcohol consumption, menopausal status, and opposing biomarker (IGF-1 or IGFBP-3)

*Statistically significant

We did not find evidence of multiplicative interaction between elevated risk score and elevated IGF-1 (Table 2); each model showed increased risk, with roughly equivalent point estimates. Modest synergistic interaction was evident in women with high IGFBP-3 and high BOADICEA (OR = 3.47, 95% CI = 1.04–11.6) compared to women with low IGFBP-3 and low BOADICEA risk. This synergistic effect was stronger than the risk for women with high IGFBP-3 and low BOADICEA compared to low IGFBP-3 and low risk (OR = 2.08, 95% CI = 0.55–7.92) and women with low IGFBP-3 and high BOADICEA compared to low IGFBP-3 and low risk (OR = 2.26, 95% CI = 0.70–7.25) (Table 2). Neither the IGF-1 nor IGFBP-3 models demonstrated significant RERI (Table 2).

## Discussion

We found an overall increased breast cancer risk for IGF-1 or IGFBP-3 elevation, although it was not statistically significant (≥ median compared to <: IGF-1 OR = 1.37, 95% CI = 0.66–2.85; IGFBP-3 OR = 1.62, 95% CI = 0.81–3.24). The magnitude of these associations is consistent with results from pooled analyses comparing women with the highest and lowest biomarker concentrations [2]; a pooled analysis of 17 prospective studies indicated a strong positive association between IGF-1 and breast cancer (*p* trend < 0.0001), with an OR of 1.28 (1.14–1.44) for women in the highest IGF-1 quintile [2]. Increased breast cancer risk was also associated with IGFBP-3 elevation in this pooled analysis (*p* trend = 0.062), with an OR of 1.13 (0.99–1.28) at the highest IGFBP-3 quintile [2]. In the analyses restricted to women with a first-degree relative with breast cancer, the risk increased to an OR of 1.62 (1.09–2.39) for women at the highest IGF-1 quintile and OR = 1.35 (0.92–1.99) for the highest IGFBP-3 quintile, further supporting the hypothesis that family history may be an important modifier [2]. We see similar increases in risk due to IGFBP-3 elevation alone and increased familial risk alone, and the combined effect of both was statistically significant (OR = 3.47, 95% CI 1.04–11.6 for high IGFBP-3 and high BOADICEA).

Study limitations include the cohort sample size; despite the consistency of our effect estimates with prior literature, we had low power, particularly for subgroup analyses. None of the women in this sample had IGF-1 or IGFBP-3 levels outside the normal range; there were therefore no clinically defined thresholds to indicate abnormal elevation. In addition, we obtained a single baseline measure of IGF-1 and IGFBP-3. Intra-individual variability exists for IGF-1, and diet can modify non-fasted IGFBP-3 levels; single biomarker measures should be interpreted with caution [10].

The results of this study offer some support that blood biomarkers, particularly IGFBP-3, may be useful in improving risk stratification, especially if replicated in larger enriched cohorts [9, 11–13]. There are fewer data on biomarkers for women at elevated breast cancer risk; these results suggest that elevated levels of IGF-1 and IGFBP-3 may be similarly related to breast cancer risk on a relative scale irrespective of absolute baseline risk. Given the higher absolute risk of breast cancer, the similar relative magnitude of risk may translate into a greater absolute risk difference from changes in these biomarkers.

## Data Availability

The datasets used and/or analyzed during the current study are available from the corresponding author on reasonable request.

## Abbreviations

BCFR: Breast Cancer Family Registry
BMI: Body mass index
BOADICEA: Breast and Ovarian Analysis of Disease Incidence and Carrier Estimation Algorithm
CI: Confidence interval
CLIA: Chemiluminescent immunoassay
CV: Coefficient of variation
ELISA: Enzyme-linked immunosorbent assay
FRP: Familial risk profile
IGF-1: Insulin-like growth factor 1
IGFBP-3: Insulin-like growth factor binding protein 3
RERI: Relative excess risk due to interaction
